# Epidemiology and Clinical Characteristics of Older Patients Transferred from Long-Term-Care Hospitals (LTCHs) to Emergency Departments by a Comparison with Non-LTCHs in South Korea: A Cross-Sectional Observational Study

**DOI:** 10.3390/ijerph19148879

**Published:** 2022-07-21

**Authors:** Soon Young Yun, Ji Yeon Lim, Eun Kim, Jongseok Oh, Duk Hee Lee

**Affiliations:** 1 National Emergency Medical Center, National Medical Center, 245, Eulji-ro, Jung-gu, Seoul 04564, Korea; ysy772@gmail.com; 2 Department of Emergency Medicine, College of Medicine, Ewha Womans University, Anyangcheonro 1071, YangChoengu, Seoul 07985, Korea; beautifuldoctor@gmail.com (J.Y.L.); silver215@hotmail.com (E.K.); 3 Department of Economics, Seoul National University, Room 208, Bld 16, 1 Gwanak-ro, Gwanak-gu, Seoul 08826, Korea

**Keywords:** emergency department, older, long-term-care hospitals, patient transfer

## Abstract

The South Korean population is rapidly aging, and the number of older patients in long-term-care hospitals (LTCHs) continues to increase. This study aims to investigate the epidemiologic data, characteristics, and clinical outcomes of patients aged 65 years and older transferred from LTCHs to emergency departments (EDs). This is a retrospective study based on National Emergency Department Information System data from 2014 to 2019. Of the 6,209,695 older patients visiting EDs for disease treatment, 211,141 (3.4%) were transferred from LTCHs. Among patients from LTCHs (211,141), 24.2% were discharged from EDs, 43.0% were admitted to general wards, 20.7% were hospitalized in intensive care units, 3.1% were transferred to another hospital, 6.1% returned to LTCHs, and 2.1% died in EDs. ED stays were the longest for those returning to LTCHs (710.49 ± 1127.43 min). Foley catheterization (40.3%) was most frequently performed in preventable ED visits. In South Korea, older patients being discharged from the ED or returning to LTCHs, after being transferred from LTCHs to EDs, increased. ED stays among older LTCH patients were longer than among non-LTCH older patients, contributing to congestion. To reduce avoidable transfer to EDs from LTCHs, it is necessary to discuss policies, such as expanding appropriate medical personnel and transitional treatment.

## 1. Introduction

The care of older patients is currently one of the most discussed topics worldwide. Many countries have already entered or are entering a super-aged society. Japan had the world’s highest proportion of a population aged 65 years and older at 23% in 2009 [1], while in 2015, China had approximately 144 million people aged 65 years and older, accounting for 10.5% of the total population. The number of people aged 65 years and older in China is projected to increase by more than two-fold to 366 million (26.1%) by 2050 [2]. The Korean society is rapidly aging, and the Statistics Korea departments estimate that the people aged 65 years and older will constitute 26.7% of the total population by 2035, increasing from just 13.4% in 2020 [3].

According to one long-term-care report from the European Union (EU-22) that assessed nationwide averages, 30.9% of people aged 65 years and older were in need of long-term care (based on 2019 data) [4]. The societal demand for medical institutions providing long-term treatment for older patients with chronic diseases is increasing. Each country has different systems related to the allocation of nursing beds. While the United States and Japan have promoted community-oriented policies for caregivers, South Korea has implemented policies to promote the supply and use of nursing beds [5]. As a result, there were only 19 long-term-care hospitals (LTCHs) in 2000, but this number climbed to 1582 in 2019 in South Korea. In addition, the number of individuals using LTCHs also increased, from only 27,000 in 2000 to 210,284 in 2021, representing a 7.8-fold increase [6]. Even after being admitted to LTCHs, however, patients are often transferred to the emergency departments (EDs) of other medical institutions according to their health status and the medical capacity of the LTCHs. After receiving treatment, many patients return and are repeatedly re-admitted to LTCHs [7].

In several European countries, studies have been conducted on the appropriate transfer of LTCHs or nursing-home patients to EDs for treatment [8]. A substantial amount of research has also been conducted on hospitalizations among LTCH patients in the United States [9]; from 2001 to 2009, annual United States ED visits by older patients increased by 24.5% from 15.9 million to 19.8 million. A notable finding was the increase in resource intensity of ED visits by older adults [10]. In addition, studies on LTCH patients’ use of hospitals and EDs have also been conducted in Canada [11]. Older patients with chronic diseases have a high need for ED resources and stay longer in the ED, which influences congestion in the ED [12]. A critical issue in Korea is the declining quality of care in LTCHs due to the rapid increase in long-term-care facilities and the number of patients seeking treatment [13]. The development of policies to address growth in the residential (institutional) sector, slow development of home and community-based services, shortages of long-term caregivers, weak quality regulations, and a lack of organized funding is underway in other countries [2].

In South Korea, the number of LTCHs has been increasing, but few studies have evaluated the patients who are transferred from LTCHs to EDs. Therefore, we aim to identify the epidemiological characteristics and clinical outcomes of older patients being transferred from LTCHs to EDs in comparison to non-LTCH older patients in South Korea. We hope that this study will provide basic data that could be used to establish policies related to LTCHs and identify the factors associated with preventable transfers.

## 2. Materials and Methods

### 2.1. Setting and Data Collection

This study was retrospectively performed using data collected from the National Emergency Department Information System (NEDIS) from January 2014 to December 2019. The NEDIS was established in 2003 to provide the basis for research and policy development related to the emergency medical system in South Korea. The NEDIS is a data system established in 2001 that transmits information to the National Emergency Medical Center’s server, which is operated under the supervision of the Ministry of Health and Welfare of Korea. Patient information is transferred automatically in real-time from all Korean EDs nationwide.

In 2019, there were 38 regional emergency medical centers (level 1), 124 local emergency medical centers (level 2), and 240 local emergency medical institutions (level 3) in Korea, and 399 out of the 402 emergency medical institutions participated in NEDIS data collection. The numbers of emergency room beds in emergency medical institutions by year were 7044 in 2014, 7099 in 2015, 7180 in 2016, 7064 in 2017, 6945 in 2018, and 7105 in 2019 [14].

**Figure 1:**
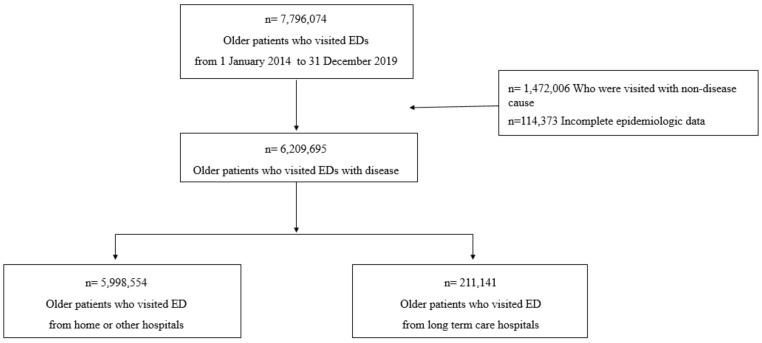
Flowgram of patients.

This study used data from all EDs nationwide in South Korea. We included all patients aged 65 years and older who visited the ED from 2014 to 2019. From 2014 to 2019, the number of older patients who visited EDs was 7,796,074, of whom 6,209,695 patients who visited the EDs for treatment related to a disease were finally enrolled (1,472,006 who visited the EDs for a non-disease cause and 114,373 with incomplete data were excluded). Of these patients, 5,998,554 visited EDs from home or other hospitals, while 211,141 were transferred from LTCHs to EDs (Figure 1).

### 2.2. Variables and Outcome Measures

The NEDIS collects the following demographic and clinical data: age, sex, date of ED visit, time of ED visit, geographic location of the ED, insurance type, means through which the ED was visited, the route of visitation (e.g., direct visit, via transfer, outpatient request, or other), whether the visit was or was not disease-related, the Korean Triage Acuity Scale (KTAS) level (KTAS was developed in 2012 by adapting the Canadian Triage and Acquisition Scale, a Canadian emergency patient triage tool, to the medical environment in South Korea) [15], the level of consciousness of the patient in the ED (alert mental state, verbal response, pain response, and unresponsiveness), the systolic and diastolic blood pressure, pulse and respiratory rates, the diagnosis of the patient in the ED, and the duration of the ED stay. The time of the ED visit was further classified as being during business or non-business hours. Urban areas included eight cities and their metropolitan areas, while rural areas included seven provinces and Jeju Island.

In this study, using variables collected in the NEDIS, older patients transferred from LTCHs and those not from LTCHs were compared. The disposition after receiving ED care (discharge, transfer to another hospital, admission to a general ward (GW) or intensive care unit (ICU), or death), and treatments and procedures received in the ED were the primary outcomes. For admitted patients, data related to the final diagnosis, duration of hospitalization, medical outcomes at the time of discharge, whether the patient was transferred to another hospital, and whether death occurred were the secondary outcomes in this study.

The groups of patients who were discharged or returned to the LTCH were combined to form what was defined as the “preventable” group [16]. Diagnoses were categorized according to the International Classification of Diseases, Tenth Revision [17].

### 2.3. Statistical Analysis

The frequency and percentage or the mean and standard deviation were reported for all variables, as appropriate. Among the descriptive variables, categorical variables were analyzed using chi-squared tests, whereas non-categorical variables were compared using Student’s *t*-tests or ANOVA, as appropriate. The variables with two-tailed *p*-values less than 0.05 were considered statistically significant and are listed in the tables. All statistical analyses were performed using IBM^®^ SPSS^®^ Statistics version 26.0 (IBM, Armonk, NY, USA).

## 3. Results

**Figure A1:**
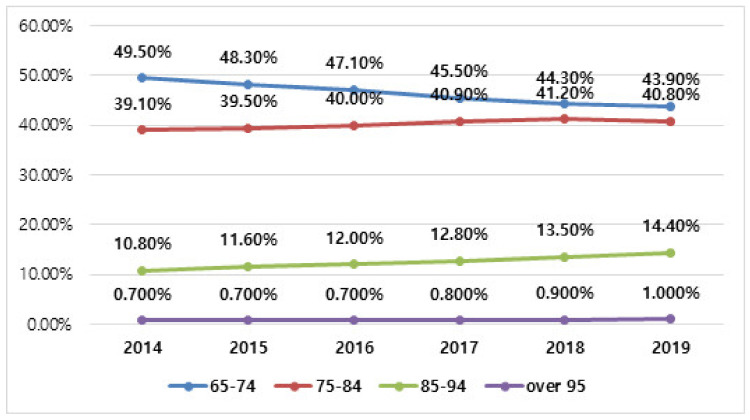
The annual number of elderly individuals who visited EDs in each 10-year age bracket from 2014~2019.

From 2014 to 2019, 6,209,695 older people visited the ED for disease-related treatment, with the annual numbers gradually increasing to 747,717 in 2014, 776,179 in 2015, 852,167 in 2016, 989,373 in 2017, 1,294,034 in 2018, and 1,550,225 in 2019. In addition, when older individuals 65 years and older were stratified into 10-year age brackets, the proportion of older people showed an increasing trend (Figure A1).

### 3.1. Epidemiological and Clinical Characteristics of Older Patients Who Were Admitted to Emergency Departments

Among the patients who visited the EDs, those who visited the ED from an LTCH accounted for 3.4% (*n* = 211,141). The mean age of those who visited the EDs from the LTCHs was 79.7 ± 7.0 years, older than non-LTCH older patients (*p* = 0.000). In the LTCH group, those aged 75 to 84 years were the most dominant (51.1%), and those over 85 years old accounted for 25.4%. The proportion of male patients was 42.0% in the LTCH group, which was statistically lower than that in the overall older group.

Of the total visits, level 1 regional emergency medical center visits of LTCH patients constituted 37.1%, higher than that of the non-LTCH older patients group (27.5%).

The levels of consciousness were significantly different between the LTCH and non-LTCH older patients: alert, 70.7% vs. 91.3%; verbal, 11.8% vs. 2.6%; pain response, 1.8% vs. 2.3%; and unresponsiveness, 15.7% vs. 3.9%.

**Figure A2:**
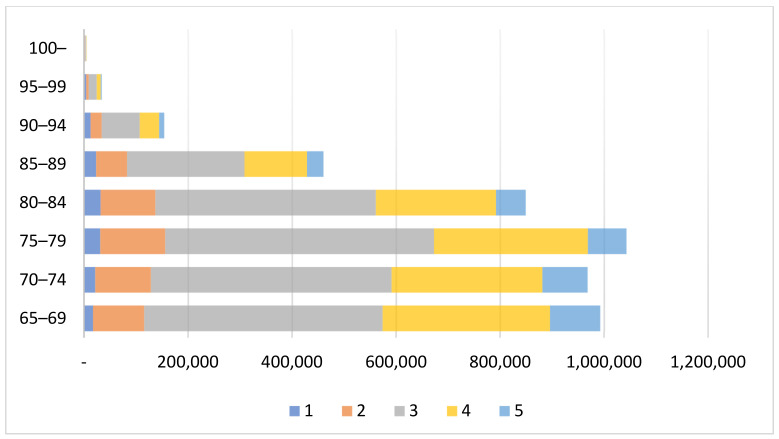
The number of elderly patients in each five-year age bracket with each Korean Triage and Acuity Scale (KTAS) score (1–5).

The severity of the KTAS increased with age. The LTCH group had more severe triage score (KTAS 1~3) than the non-LTCH group (Figure A2).

In the EDs, the LTCH group had a higher proportion of consultations for pulmonology (14.9%), nephrology (11.1%), and infection (3.9%) than the non-LTCH group.

The length of the ED stay was 344.18 ± 682.2 min for the non-LTCH older patients group, compared to 572.38 ± 856.2 min for the LTCH group. Compared to the non-LTCH older patients group, the LTCH group had a higher rate of hospitalization to the ward and ICU, and the rate of being transferred back to the LTCH after ED treatment was also higher. The rate of ED deaths was similar between the two groups (Table 1).

### 3.2. Comparison of Groups According to ED Results of Patients Transferred from Long-Term-Care Hospitals

The data from 209,763 older individuals visiting EDs from LTCHs were analyzed. Of these patients, 51,176 (24.4%) were discharged from the ED, 90,879 (43.3%) were admitted to a GW, 43,626 (20.8%) were hospitalized in an ICU, 6642 (3.2%) were transferred to another hospital, 12,952 (6.2%) were returned to an LTCH, and 4488 (2.1%) died in the ED. Those aged 65–74 years accounted for 23.5% of all cases, those aged 75–84 years accounted for 51.1%, and those aged 85 years older accounted for 25.4%.

**Figure 2:**
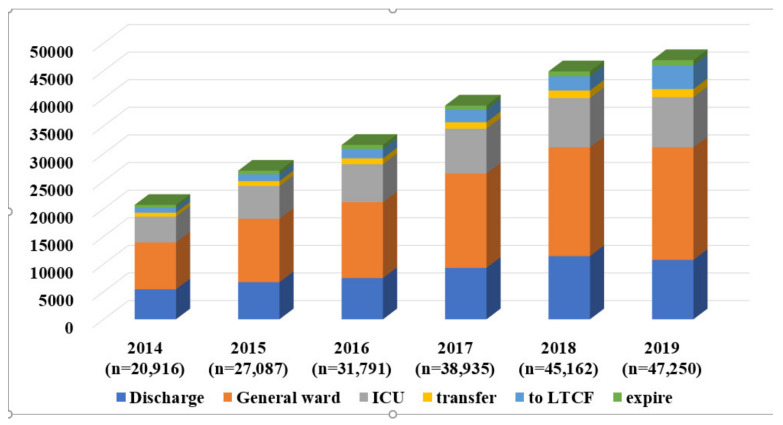
The annual number of older patients visiting the ED from LTCHs and the clinical outcomes. ED, emergency department; ICU, intensive care unit; LTCHs, long-term-care hospitals.

From 2014 to 2019, the number of older patients who were transferred from LTCHs increased annually, and the outcomes of the ED treatments are shown in Figure 2.

There were 64,128 patients in the preventable group, accounting for 30.6% of the older patients from LTCHs.

Among those whose visits were classified as preventable, 38.4% of those in the discharged group and 41.4% of those in the group who returned to LTCHs had visited regional emergency medical centers. In terms of the time of day, 62.1% of patients from LTCHs visited EDs during business hours while 42.8% of non-LTCH patients visited ED during business hours. Overall, the level of consciousness was classified as alert 70.7%, verbally responsive 11.8%, pain responsive 1.8%, and unresponsive 15.7%. The proportion of patients with poor consciousness who were not classified as alert was high in the death (73%) and the ICU admission (45%) groups (*p* = 0.000). The duration of the ED stay was longest for those who returned to LTCHs, at 710.49 ± 1127.43 min (Table 2).

### 3.3. Comparison of the Length of Hospitalization and Outcomes between Older Patients Not from LTCHs and from LTCHs

Among the older patients visiting the EDs who did not come from LTCHs, 1,949,871 (32.5%) were admitted to GWs and 503,300 (8.4%) to ICUs. These numbers increased steadily from 2014 to 2019. Patients admitted to a GW stayed in the hospital for 261.23 ± 360.16 h and those admitted to the ICU stayed for 375.95 ± 514.52 h. Of the patients hospitalized in a GW, 2.4% returned to the LTCHs and 5.8% died, whereas among patients admitted to ICU, 6.2% returned to LTCHs and 17.3% died.

Among the older patients visiting EDs from LTCHs, specifically, 90,879 (43.0%) were admitted to GWs and 43,626 (20.7%) to ICUs; these numbers increased steadily from 2014 to 2019. Patients admitted to a GW were hospitalized for 365.76 ± 476.72 h, whereas those admitted to an ICU were hospitalized for 443.36 ± 565.36 h. Of the patients hospitalized in a GW, 26.2% returned to the LTCH and 13.4% died; among those hospitalized in an ICU, 29.9% returned to the LTCH and 26.1% died (Table 3).

**Figure 3:**
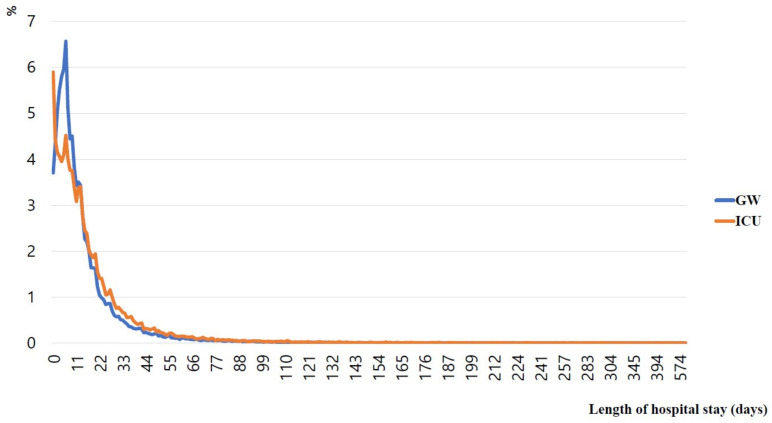
Proportion (%) of hospitalized (ICU or GW) patients who visited the emergency department from LTCHs by duration of hospitalization in days. GW, general ward; ICU, intensive care unit; LTCHs, long-term-care hospitals.

The proportion of patients with a hospitalization period of 7 days or less was 54.8% for those admitted to GWs and 46.0% for those admitted to ICUs; the values were 87.9% for those in GWs and 82.7% for those in ICUs among patients with a 30-day hospital stay (Figure 3).

### 3.4. Comparison of the Chief Complaints between Discharged and Admitted Patients Transferred from LTCHs

The chief complaints of the discharged group were dyspnea in 4455 (8.7%), fever in 3753 (7.3%), catheter care in 2786 (5.4%), and abdominal pain in 2747 (5.4%) cases. The chief complaints among the group returning to LTCHs were dyspnea in 1860 (14.4%), fever in 1512 (11.7%), and abdominal pain in 740 (5.7%) cases. For those admitted to GWs, the three most common chief complaints were dyspnea in 18,212 (20.0%), fever in 17,787 (19.6%), and gastrointestinal bleeding in 6475 (7.1%) cases. For those admitted to ICUs, the most common chief complaints were dyspnea in 13,420 (30.8%), fever in 5731 (13.1%), and gastrointestinal bleeding in 2925 (6.7%) cases (Table 4).

### 3.5. Diagnosis Analysis of Older Patients from LTCHs

Among the older patients transferred to the EDs from LTCHs, 41,588 (19.8%) had severe illness diagnosis code. Table 5 shows the ratio of patients with severe disease by disposition results in EDs. A total of 34.8% of the death group and 32.0% of the ICU admission group had severe illness diagnosis codes, whereas 9.1% of the discharged group and 12.2% of the return to LTCHs group had severe illness diagnosis codes. Surgical emergencies, cerebral infarct, sepsis, and acute renal failure were the most common severe illness diagnosis groups in the LTCH group admitted to an ICU and GW.

The diagnoses were compared among patients who were transferred from LTCHs to EDs (Table A1). The main diagnoses were infection-related diseases, such as pneumonia, urinary tract infection, fever, and sepsis.

### 3.6. Procedures Performed for LTCH Patients Who Visited EDs

A total of 127,931 procedures were performed in the ED for patients transferred from LTCHs. In the non-preventable group, central venous catheter insertion (39.5%) was the most frequently performed procedure. In contrast, in the preventable group, Foley catheterization (40.3%), central venous catheterization (12.6%), and nasogastric tube insertion (9.1%) were the most frequently performed procedures (Table 6).

## 4. Discussion

This study analyzed nationwide ED data to determine the degree of ED use among patients from all LTCHs in Korea. Previously, a single-center study was published on this topic, although it assessed the hospitalization period of transferred patients, not the use of EDs, specifically [18]. In this study, it was found that transfers from LTCHs to EDs continued to increase from 2014 to 2019.

We investigated the outcomes and characteristics of visits to EDs among patients from LTCHs. This study also made it possible to determine the main symptoms reported by patients and which treatments were being performed the most often. Although a limitation of this study is that it did not evaluate the patients’ histories and usual conditions, it still provides very important basic data about this growing patient population. It is difficult to accurately determine the appropriateness and inadequacy of treatments due to limitations related to the fact that the evaluation of the hospital staff was not performed in real-time. However, by investigating the treatment received by many patients who returned to LTCHs or were discharged from EDs, this study clearly demonstrates that the quality of care and possible treatments available at LTCHs are insufficient.

The Organization for Economic Co-Operation and Development (OECD) defines “long-term-care beds in hospital” as a hospital bed for patients in need of long-term care due to reduced independence in daily life as a result of chronic diseases and functional disorders [19]. The scope and definition of long-term-care beds in hospitals in countries that have submitted data to the OECD can vary greatly from country to country, although the definitions can be broadly classified into several types as follows. The first is for countries that include palliative care beds as long-term-care beds, including Belgium, Denmark, and Iceland. The second is for a country that includes long-term-care beds in geriatric hospitals or general hospitals, such as in Belgium, France, Iceland, and Sweden. Finally, Canada, Poland, and the United States are countries that have institutionalized LTCHs in the form of beds rather than independent hospitals [20,21,22].

In South Korea, there were only 19 LTCHs at the end of December 2000, but 1585 at the end of October 2020; this change represents an approximate 83-fold increase over 20 years. LTCHs are mainly used by patients who need long-term care or those with chronic diseases. Depending on the change in their health status or the capacity of the hospital, patients admitted to LTCHs are transferred to other medical institutions, but some of these patients are re-admitted to LTCHs repeatedly. The number of long-term-care beds per 1000 people aged 65 years and older in South Korea was 36.7 as of 2017, which is more than 10-times higher than the national average among OECD countries of 3.6 [7]. In South Korea, this trend is increasing, unlike in other countries (such as Japan, France, United States, and Sweden) that are experiencing a declining trend. Over a period of 10 years (2008–2018), the number of general hospital beds, excluding long-term-care beds, has not changed much, whereas the number of long-term-care beds in long-term-care hospitals has risen sharply, with a 255.4% increase in Korea [7].

The problem with Korean LTCHs is that the quality of medical care is declining due to the rapid increase in the quantity of patients seeking treatment. Due to the characteristics of patients from nursing hospitals, common problems among older individuals, those with dementia, and bedridden patients include communication difficulties and challenges in diagnosing diseases [23,24]. Older patients, especially those with infectious diseases, presented with many non-specific symptoms, and there is a shortage of medical staff specializing in geriatric medicine, as well as limitations on tests that are required for diagnosis. Of the 694,695 medical personnel in South Korea, 342,455 (49%) are employed in general hospitals and only 83,480 (12%) are in LTCHs [13].

To improve the quality of medical care, skilled nursing personnel are being recruited, although there is a chronic shortage of nursing personnel. Other problems include the lack of financial resources and the resulting wage gap in acute hospitals, the lack of specialized education related to care of the elderly, and the lack of continuity in patient management due to frequent turnover [23].

It is known that the older adults are generally more likely to have chronic diseases [25], and those with chronic diseases are more likely to use EDs [26]. ED visits by chronically ill patients remain a key cause of overcrowding. If the duration of stay in the emergency room is prolonged due to such overcrowding, the death rate during hospitalization increases by 4.3% to 5.8%. There was a linear relationship between the Overcrowding Hazard Scale and deaths on day 7. In the case of patients 50 years or older, the hazard ratio was 3.3 [27]. Our study found that the ED length of stay was longer in LTCH patients than in non-LTCH patients, which could affect the congestion of the EDs. The occupation of emergency medical resources and an increase in medical consumption by chronically ill patients can overload emergency medical services and affect the treatment capacity for patients with severe disease-related emergencies. Therefore, it is necessary to devise a plan for the appropriate treatment of chronically ill patients in LTCHs. In this study, the ED stay was longest for patients returning to LTCHs, at 710.49 ± 1127.43 min. This is a factor that influences the congestion of EDs, and a longer stay in the ED is adversely related to patients’ clinical outcomes [12]. ED crowding can be defined as a situation in which the demand for emergency services exceeds the ability of physicians and nurses to provide quality care within a reasonable period. In the present study, LTCH patients visited the regional emergency medical centers, which are level 1 (for most critical patients) emergency centers. Frequent visits to the regional emergency medical centers by LTCH patients exacerbate ED congestion.

Preventable transfers from LTCHs to EDs can also cause ED crowding, which is a major problem afflicting healthcare systems. Grant et al. reported that interventions involving multidisciplinary care teams and/or regular visits by providers were the most effective means of reducing preventable transfers to the ED from LTCHs [28]. This may be because it requires a lot of time to treat older patients in general as well as older patients with various diseases from LTCHs, or this may be a result of the medical staff lacking the necessary skills. Another possible explanation is the delay in decision making related to treatments or disposition. Some studies have shown that many ED physicians and nurses have not been well-trained in geriatric emergency medicine and felt less comfortable when meeting older patients [29,30]. As the older population increases, however, it is becoming increasingly necessary to reconsider how the medical care system is designed to provide treatment for the older patients in the ED. Additional education on geriatric care should also be provided [31].

Gruneir et al. reported that 23% of patients visited the ED at least once during a six-month period, and one quarter of all visits was deemed to have been preventable. They showed that trauma was the leading cause of transfer among patients from LTCHs, occurring in 21.5% of cases, and trauma was not included as a factor in the preventable visit [11]. However, this study excluded trauma patients because it was impossible to determine whether trauma was the inevitable cause of transfer from LTCHs due to insufficient diagnostic resources. In this study, 62.1% of patients from LTCHs visited EDs during business hours, indicating that LTCHs are insufficiently equipped to evaluate or treat patients, even during business hours.

The mortality rate in the EDs among patients who visited from LTCHs was 2.1%, similar to that of the older patients not from LTCHs (Table 1). However, the ICU admission rate was higher in LTCH patients than in the non-LTCH group, and the mortality rate after ICU admission was also higher in LTCH patients than in the non-LTCH group (26.1% versus 17.3%, respectively). This is considered to largely be a result of limitations of the system and patients’ families related to decisions on whether to provide life-sustaining treatment in LTCHs. Patients often experience burdensome and unnecessary treatment transitions, especially until the end of life. Providers understand that families play an important role in ED-transfer decisions, as families often respond to changes in a resident’s condition, such as when a family member is in crisis. Stephens et al. reported that the four main causes for this were instability in LTCHs, the lack of preparation for death among patients’ family members, a lack of or inadequate advance treatment planning, and a lack of communication and agreement among family members about treatment goals [32].

In Korea, the Act on Hospice and Palliative Care and Decisions on Life-Sustaining Treatment for Patients at the End of Life was enacted in 2018, and the number of registrations of “Advance statement on life-sustaining treatment (Advance Directive)” increased sharply, with 532,667 registered cases as of December 2019. At the same time, there were 80,003 cases of withholding or withdrawal of life-sustaining treatment, of which only 1322 cases (1.7%) were fulfilled by advance directive. In addition, the number of cases of withholding or withdrawal of life-sustaining treatment in LTCHs was very small, with 328 cases (0.4%) [33]. Of note, a previous study showed that the proportion of Advance Directives in withholding or withdrawing life-sustaining treatment was only 1.37% [34]. As a supplement to this, a policy framework should be provided to guide decision making by LTCHs with regard to life-sustaining treatment in South Korea. Through financial or human resource support, life-sustaining treatment decisions can be made at the LTCHs, reducing unnecessary transfers to the EDs.

Chou et al. conducted a study on the transfer to EDs from LTCHs in Taiwan, where LTCHs had primary care physicians, such as in Korea. The common causes of ED transfer were infection (23.5%), fall-related injury (7%), gastrointestinal problem (9.9%), and altered mental state (9.3%). In addition, onsite primary care geriatricians may play an important role in such settings [11,35]. In this study, dyspnea, fever, abdominal pain, and gastrointestinal bleeding were common in both the discharged and the hospitalized groups. As a result of the severity analysis at the time of admission, severe patients had symptoms, such as decreased consciousness, fever, and cardiac arrest from complications related to comorbidities. However, the main symptom of mild patients was catheter replacement or tube replacement. In group of returning to LTCH at ED, some cases reported catheter care or hematuria as the chief complaint. Although it cannot be concluded that all cases of hematuria were catheter-related, 40.3% of the patients who were discharged or re-admitted from the ED received foley catheterization, suggesting that there were many visits due to catheter-related problems.

In this study, Foley catheterization, central venous catheter insertion, and nasogastric tube insertion were the most frequently performed treatment procedures in the group discharged from an ED (discharge group and return to LTCHs). As mentioned earlier, the number of LTCHs with primary care physicians has increased in Korea. Our study was about the transfer of older patients to EDs from LTCHs with attending primary care physicians. It is thought that quality control by the onsite attending physician can ensure adequate transfer of patients and avoid unnecessary transfers. In a six-year national study to identify preventable ED visits by nursing home residents in the United States, 1.8% of visits of nursing homes were made by nursing home seniors. More than half (53.5%) of their ED visits did not lead to hospitalization, and of the patients discharged from the ED, 62.8% had normal vital signs and 18.9% received no diagnostic testing prior to discharge from the ED [36]. In the present study, patients from LTCHs accounted for 3.4% of all older patients, and 30.3% of them were discharged from the ED. Non-emergency (KTAS 4, 5) patients comprised 21.6% of the cases. Of course, this does not mean that all patients discharged from the ED or returning to LTCH represent preventable cases, and even some hospitalizations can be preventable.

Based on 77 studies, Lemoyne et al. reported that 4–55% of ED visits by LTCH residents were inappropriate. However, the definition of relevance is not uniform across studies and warrants further investigation. To avoid inappropriate transfers to the ED, the authors recommended respect for patient autonomy, adequate nurse staffing, training of nurses, and the enhanced role of physicians’ in LTCHs [8].

We agree with a review study on avoidable ED transfers among patients coming to the ED from LTCHs [11], which concluded that access to EDs is an important factor in the quality of care for patients in long-term-care institutions. It suggests that preventable power sources exist and have defined in several studies, although it argues against the standard proposed as an appropriate power source. From a clinical point of view, there is still no consensus on what is truly avoidable and preventable. The main trends relate to reforms, such as improving the welfare of informal caregivers; promoting accessibility, affordability, and quality of home care services and residential care; and improving the welfare of skilled long-term-care workers [4]. In addition, to prevent the unnecessary transfer or repeated visits to the ED, it is necessary to establish an integrated information system and expand the emergency medical system. Despite the distinct differences between national systems regarding the characteristics of the aging population, countries face common challenges in the field of long-term care. These include the challenge of providing affordable access to long-term-care services to anyone in need, the problem of providing quality long-term-care services, issues of securing an adequate long-term-care workforce with good working conditions, and supporting informal caregivers. Notably, healthcare systems are grappling with the problem of financing long-term care at a time when demand for care is rising [4].

According to an analysis of patients visiting the ED in 2020, the number of older patients with mild symptoms was higher than that of patients with severe symptoms compared to the previous year [37]. One of the reasons is that relatively simple procedures cannot be performed in the LTCHs, and there is a need to alleviate the risk of infection. In Korea’s medical system, LTCHs have practical limitations, but their social roles are clear and they play a major role in the treatment of older patients and chronically ill individuals. Policy efforts, such as strengthening LTCH requirements, fostering the training of professional nurses and nursing assistants, and providing adequate human resource support, are necessary.

The coronavirus disease (COVID-19) pandemic has exposed older people to health and social risks as a consequence of other diseases and decreased function [38]. In the ongoing COVID-19 situation, LTCHs can provide acute care to the residents and reduce the need for hospital transfer. A recent study highlighted the need for a virtual centralized hub to facilitate the provision of acute care to residents with emergency medical problems in LTCHs and reduce the need for hospital transfers in a COVID-19 context [39]. Such a treatment system can be necessary in Korea as well. Before devising such a plan, it is necessary to first analyze the kind of treatment interventions that are currently being provided in the LTCHs. However, prior research that promoted the classification of specialized treatment fields in LTCHs in the United States [40] did not clearly analyze the characteristics of about 60% of the hospitals. Therefore, in addition to the need for further data analysis, a detailed review of policy requirements should be performed.

## 5. Limitations

This study has several limitations. In the case of NEDIS data, the patient was not personally identified and treatment was provided anonymously at each visit. Therefore, it was difficult to ascertain whether a single patient has made several visits to the ED. Although this study had the advantage of using nationwide data, the exclusion of 114,373 patients (1.3% of all patients aged 65 years and above) because of incomplete data might have biased the findings.

Consciousness was significantly altered in the ICU admission and death groups. Future research should evaluate not only the level of consciousness at the time of admission to the ED, but also whether it has changed from the usual. It remains unknown whether the patient’s discharge and re-transfer was a solution to the problem, whether it was hopeless discharge in a do-not-resuscitate situation, or whether there was a decision to discontinue life-sustaining treatment. We analyzed only patients who visited the ED, and it was not possible to know how many outpatient visits were from LTCHs. However, it would be meaningful to analyze patients who visited the ED because of mobility-related issues or the need to undergo a procedure. Only patients who were transferred from LTCHs were analyzed. South Korea has a large number of LTCHs, which is thought to be meaningful, but characteristics of the individual nursing homes (without doctors, only nurses) cannot be analyzed due to a lack of such data. As the number of nursing homes grows along with the increasing number of older patients and LTCHs, it is also necessary to analyze data of patients in nursing homes.

## 6. Conclusions

In an aging Korean society, the number of older patients hospitalized in LTCHs is rapidly increasing, as is the number of older patients being transferred to EDs for treatment. An increasing number of patients are also being discharged from EDs or returning to LTCHs. The length of stay in the ED among patients from LTCHs was longer than that of the non-LTCH older patients, which may contribute to increased congestion in EDs. The ability to reduce the number of avoidable transfers to the ED through the expansion of appropriate medical personnel and transitional care services offered in LTCHs would help and could also reduce the discomfort experienced by patients. LTCHs play an important societal role in the treatment of older patients and those that are chronically ill; therefore, it is important to develop policies to facilitate improved care. Providing a broader policy agenda requires ongoing discussions on the treatment and maintenance of patients from LTCHs and ways to minimize preventable ED visits.

## Figures and Tables

**Table 1:** Epidemiological and clinical characteristics of older patients who were admitted to EDs.

	Patients Who Visited ED Not from LTCHs		Patients Who Visited ED from LTCHs		Total		*p*-Value
	*n*	%	*n*	%	*n*	%	
	5,998,554	(96.6)	211,141	(3.4)	6,209,695	(100.0)	
Age (years, mean ± SD)	76.1 ± 7.2		79.7 ± 7.0		76.2 ± 7.3		0.000
Age groups							0.000
65–74 years	2,712,398	(45.2)	49,613	(23.5)	2,762,011	(44.5)	
75–84 years	2,477,159	(41.3)	107,802	(51.1)	2,584,961	(41.6)	
≥85 years	808,997	(13.5)	53,726	(25.4)	862,723	(13.9)	
Sex (male) [*n*(%)]	2,901,263	(48.4)	89,791	(42.5)	2,991,054	(48.2)	0.000
Level of ED							0.000
Regional emergency medical center	1,649,090	(27.5)	78,339	(37.1)	1,727,429	(27.8)	
Local emergency medical center	3,627,287	(60.5)	132,509	(62.8)	3,759,796	(60.5)	
Local emergency medical room	722,177	(12.0)	293	(0.1)	722,470	(11.6)	
Insurance							0.000
National health insurance	5,269,883	(87.9)	176,335	(83.5)	5,446,218	(87.7)	
Medicaid	629,684	(10.5)	28,785	(13.6)	658,469	(10.6)	
Others	98,987	(1.7)	6021	(2.9)	105,008	(1.7)	
Visiting means							0.000
911	1,686,820	(28.1)	5902	(2.8)	1,692,722	(27.3)	
Other ambulance	563,114	(9.4)	163,261	(77.3)	726,375	(11.7)	
Car	3,639,159	(60.7)	40,642	(19.2)	3,679,801	(59.3)	
Walk	78,374	(1.3)	202	(0.1)	78,576	(1.3)	
Others	31,087	(0.5)	1134	(0.5)	32,221	(0.5)	
Consciousness							0.000
Alert	4,831,704	(91.3)	149,275	(70.7)	4,980,979	(90.5)	
Verbal	137,663	(2.6)	24,906	(11.8)	162,569	(3.0)	
Pain response	119,123	(2.3)	3885	(1.8)	123,008	(2.2)	
Unresponsiveness	204,664	(3.9)	33,062	(15.7)	237,726	(4.3)	
KTAS							0.000
Level 1	136,478	(3.1)	8136	(5.0)	144,614	(3.2)	
Level 2	490,436	(11.3)	31,519	(19.3)	521,955	(11.6)	
Level 3	2,089,701	(48.1)	88,197	(54.1)	2,177,898	(48.3)	
Level 4	1,277,212	(29.4)	27,283	(16.7)	1,304,495	(28.9)	
Level 5	351,196	(8.1)	7919	(4.9)	359,115	(8.0)	
Vital signs							0.000
Systolic blood pressure (mmHg)	137.41 ± 28.49		124.39 ± 28.79		136.91 ± 28.62		
Diastolic blood pressure (mmHg)	78.36 ± 15.79		72.46 ± 16.72		78.13 ± 15.87		
Pulse rate (beats/minute)	85.46 ± 19.20		90.45 ± 21.63		85.65 ± 19.32		
Respiratory rate (beats/minute)	19.87 ± 3.37		20.67 ± 4.18		19.90 ± 3.41		
Body temperature (°C)	36.76 ± 0.82		36.84 ± 0.77		36.76 ± 0.81		
Saturation (%)	96.06 ± 5.17		94.28 ± 6.49		95.96 ± 5.26		
Any abnormalities	5,060,179	(84.4)	181,355	(85.9)	5,241,534	(84.4)	
pulse ≤ 60 ≥ 100	2,241,853	(37.4)	77,188	(36.6)	2,319,041	(37.3)	
SBP ≤ 90 ≥ 180	1,474,669	(24.6)	35,963	(17.0)	1,510,632	(24.3)	
BT ≤ 36 ≥ 38.5	1,788,202	(29.8)	34,065	(16.1)	1,822,267	(29.3)	
Respiration ≥ 20	4,332,578	(72.2)	153,160	(72.5)	4,485,738	(72.2)	
Saturation < 93%	270,126	(4.5)	31,503	(14.9)	301,629	(4.9)	
Main department							
Internal medicine							0.000
Cardiology	317,558	(6.0)	11,494	(5.4)	329,052	(6.0)	
Pulmonology	305,849	(5.8)	31,484	(14.9)	337,333	(6.1)	
Gastroenterology	375,112	(7.1)	23,358	(11.1)	398,470	(7.2)	
Nephrology	149,477	(2.8)	15,408	(7.3)	164,885	(3.0)	
Endocrine	34,612	(0.7)	854	(0.4)	35,466	(0.6)	
Infection	71,370	(1.3)	8150	(3.9)	79,520	(1.4)	
Hemato-oncology	131,796	(2.5)	4597	(2.2)	136,393	(2.5)	
Allergy	1836	(0.0)	72	(0.0)	1908	(0.0)	
Rheumatology	3889	(0.1)	181	(0.1)	4070	(0.1)	
Other internal medicine	344,196	(6.5)	23,956	(11.3)	368,152	(6.7)	
Surgery							
General surgery	182,754	(3.5)	8381	(4.0)	191,135	(3.5)	
Neuro surgery	155,650	(2.9)	4724	(2.2)	160,374	(2.9)	
Thoracic surgery	38,258	(0.7)	1809	(0.9)	40,067	(0.7)	
Orthopedic surgery	68,351	(1.3)	2326	(1.1)	70,677	(1.3)	
Plastic surgery	4718	(0.1)	258	(0.1)	4976	(0.1)	
Other surgery	5689	(0.1)	148	(0.1)	4385	(0.1)	
OBGY	11,039	(0.2)	607	(0.3)	11,646	(0.2)	
Neurology	354,966	(6.7)	9910	(4.7)	364,876	(6.6)	
Emergency medicine	2,493,432	(47.1)	54,328	(25.7)	2,547,760	(46.3)	
ED length of stay (minutes, mean ± SD)	344.18 ± 682.29		572.38 ± 856.27		351.95 ± 690.17		0.000
ED outcome							0.000
Discharge	3,227,175	(53.8)	51,176	(24.2)	3,278,351	(52.8)	
Admission to GW	1,949,871	(32.5)	90,879	(43.0)	2,040,750	(32.9)	
Admission to ICU	503,300	(8.4)	43,626	(20.7)	546,926	(8.8)	
Transfer to others	173,317	(2.9)	6642	(3.1)	179,959	(2.9)	
Transfer to long-term-care hospital	11,758	(0.2)	12,952	(6.1)	24,710	(0.4)	
Expire	118,742	(2.0)	4488	(2.1)	123,230	(2.0)	
Unclassified	14,391	(0.2)	1378	(0.7)	15,769	(0.3)	

ED, emergency department; GW, general ward; ICU, intensive care unit; KTAS, Korean Triage Acuity Scale; LTCH, long-term-care hospital; OBGY, obstetrics and gynecology; SBP, systolic blood pressure; SD, standard deviation.

**Table 2:** Comparison of groups according to ED outcomes among patients transferred from LTCHs.

	Discharged		Admitted to Ward		Admitted ICU		Transfer		Return to LTCH		Death		Total		*p*-Value
	*n*	%	*n*	%	*n*	%	*n*	%	*n*	%	*n*	%	*n*	%	
	51,176	(24.4)	90,879	(43.3)	43,626	(20.8)	6642	(3.2)	12,952	(6.2)	4488	(2.1)	209,763	(100.0)	
Age (years, mean ± SD)	79.55 ± 7.17		79.75 ± 6.94		79.61 ± 6.89		79.57 ± 7.02		80.05 ± 7.22		80.33 ± 7.03		79.71 ± 7.01		0.00
Age groups															0.00
65–74 years	12,747	(24.9)	20,872	(23.0)	10,195	(23.4)	1593	(24.0)	3001	(23.2)	945	(21.1)	49,353	(23.5)	
75–84 years	25,432	(49.7)	46,897	(51.6)	22,786	(52.2)	3397	(51.1)	6341	(49.0)	2240	(49.9)	107,093	(51.1)	
≥85 years	12,997	(25.4)	23,110	(25.4)	10,645	(24.4)	1652	(24.9)	3610	(27.9)	1303	(29.0)	53,317	(25.4)	
Sex (male) [*n* (%)]	20,724	(40.5)	38,687	(42.6)	19,261	(44.2)	2982	(44.9)	5486	(42.4)	2107	(46.9)	89,247	(42.5)	0.00
Level of ED															0.00
Regional emergency medical center	19,652	(38.4)	33,609	(37.0)	15,879	(36.4)	2019	(30.4)	5362	(41.4)	1719	(38.3)	78,240	(37.3)	
Local emergency medical center	31,478	(61.5)	57,156	(62.9)	27,667	(63.4)	4593	(69.2)	7570	(58.4)	2766	(61.6)	131,230	(62.6)	
Local emergency medical institution	46	(0.1)	114	(0.1)	80	(0.2)	30	(0.5)	20	(0.2)	3	(0.1)	293	(0.1)	
Area of residence															0.00

**Table 3:** Length of hospital stay and outcomes.

	Not from LTCHs			From LTCHs		
	General Ward	ICU	*p*-Value	General Ward	ICU	*p*-Value
	1,949,871	503,300		90,879 (43.0)	43,626 (20.7)	
2014	245,577	69,097		8495	4670	
2015	255,837	69,657		11,471	5941	
2016	278,646	75,426		13,736	6884	
2017	319,336	84,812		17,073	8147	
2018	394,265	97,180		19,712	8918	
2019	456,210	107,128		20,392	9066	
Length of stay (hours, mean ± SD)	261.23 ± 360.16	375.95 ± 514.52	0.000	365.76 ± 476.72	443.36 ± 565.36	0.000
Length of stay (hours, IQR)	161.84–214.16	224.50–333.10		233.67–305.79	286.72–407.52	
Outcome after admission	*n*(%)	*n*(%)	0.000	*n*(%)	*n*(%)	0.000
Discharged	1,628,854 (86.1)	315,600 (64.8)		43,920 (49.6)	13,542 (31.9)	
Transfer to others	109,702 (5.8)	57,152 (11.7)		9538 (10.8)	5132 (12.1)	
Transfer to LTCH	44,870 (2.4)	30,040 (6.2)		23,248 (26.2)	12,667 (29.9)	
Death	109,273 (5.8)	84,167 (17.3)		11,894 (13.4)	11,050 (26.1)	

ICU, intensive care unit; IQR, interquartile range; LTCH, long-term-care hospital; SD, standard deviation.

**Table 4:** Common chief complaints of patients from LTCHs.

	Discharged			Admitted to GW			Admitted ICU			Transfer			Return to LTCH		
	51,176			90,879			43,626			6642			12,952		
		*n*	(%)		*n*	(%)		*n*	(%)		*n*	(%)		*n*	(%)
1	Dyspnea	4455	(8.7)	Dyspnea	18,212	(20.0)	Dyspnea	13,420	(30.8)	Dyspnea	1520	(22.9)	Dyspnea	1860	(14.4)
2	Fever	3753	(7.3)	Fever	17,787	(19.6)	Fever	5731	(13.1)	Fever	1020	(15.4)	Fever	1512	(11.7)
3	Care of catheter	2786	(5.4)	GI bleeding	6475	(7.1)	Mental status changes	3936	(9.0)	Mental status changes	541	(8.1)	Abdominal pain	740	(5.7)
4	Abdominal pain	2747	(5.4)	Abdominal pain	6169	(6.8)	GI bleeding	2925	(6.7)	Abdominal pain	475	(7.2)	GI bleeding	700	(5.4)
5	General weakness	1971	(3.9)	General weakness	4473	(4.9)	Hypotension	1957	(4.5)	GI bleeding	290	(4.4)	General weakness	599	(4.6)
6	Hematuria	1863	(3.6)	Mental status changes	2166	(2.4)	General weakness	1539	(3.5)	General weakness	260	(3.9)	Mental status changes	585	(4.5)
7	Chest pain	1586	(3.1)	Hypotension	1723	(1.9)	Abdominal pain	1510	(3.5)	Hypotension	221	(3.3)	Hematuria	367	(2.8)
8	GI bleeding	1439	(2.8)	Hematuria	1401	(1.5)	Chest pain	1157	(2.7)	Hematuria	101	(1.5)	Hypotension	295	(2.3)
9	Dizziness	967	(1.9)	Chest pain	1134	(1.2)	Seizure	606	(1.4)	Chest pain	69	(1.0)	Care of catheter	277	(2.1)
10	Dysarthria	644	(1.3)	Diarrhea	1166	(1.3)	Left hemiparesis	352	(0.8)	Seizures	63	(0.9)	Chest pain	211	(1.6)

GW, general ward; GI, gastrointestinal; ICU, intensive care unit; LTCH, long-term-care hospital.

**Table 5:** Percentage of patients with severe illness diagnosis codes by disposition results in EDs for LTCH group.

Severe Illness Diagnosis Group	Discharged		Admitted to Ward		Admitted to ICU		Transfer		Return to LTCH		Death	
	*n*	%	*n*	%	*n*	%	*n*	%	*n*	%	*n*	%
Acute myocardial infraction	75	(0.15)	232	(0.26)	831	(1.90)	71	(1.07)	25		75	(1.67)
Cerebral infarct	1216	(2.38)	3083	(3.39)	2109	(4.83)	192	(2.89)	322	(2.49)	16	(0.36)
Intracranial hemorrhage	177	(0.35)	237	(0.26)	755	(1.73)	153	(2.30)	90	(0.69)	31	(0.69)
Aortic dissection	22	(0.04)	22	(0.02)	61	(0.14)	20	(0.30)	10	(0.08)	16	(0.36)
Biliary diseases	405	(0.79)	2356	(2.59)	407	(0.93)	152	(2.29)	85	(0.66)	13	(0.29)
Surgical diseases	1431	(2.80)	5857	(6.44)	3182	(7.29)	326	(4.91)	536	(4.14)	176	(3.92)
Gastrointestinal bleeding/foreign bodies	53	(0.10)	345	(0.38)	173	(0.40)	10	(0.15)	18	(0.14)	4	(0.09)
Tracheobronchial bleeding/foreign bodies	116	(0.23)	319	(0.35)	105	(0.24)	24	(0.36)	35	(0.27)	8	(0.18)
Status epilepticus	17	(0.03)	70	(0.08)	178	(0.41)	8	(0.12)	5	(0.04)	3	(0.07)
Meningitis	11	(0.02)	63	(0.07)	41	(0.09)	5	(0.08)	1	(0.01)	0	(0.00)
Sepsis	122	(0.24)	1429	(1.57)	2465	(5.65)	173	(2.60)	80	(0.62)	224	(4.99)
Diabetic coma	10	(0.02)	45	(0.05)	84	(0.19)	4	(0.06)	4	(0.03)	7	(0.16)
PTE/DVT	175	(0.34)	678	(0.75)	476	(1.09)	33	(0.50)	58	(0.45)	21	(0.47)
Arrhythmia	356	(0.70)	570	(0.63)	482	(1.10)	41	(0.62)	89	(0.69)	24	(0.53)
ARDS/pulmonary edema	115	(0.22)	1079	(1.19)	1020	(2.34)	67	(1.01)	55	(0.42)	90	(2.01)
DIC	0	(0.00)	3	(0.00)	9	(0.02)	3	(0.05)	1	(0.01)	1	(0.02)
Intussusception/intestinal obstruction	59	(0.12)	385	(0.42)	121	(0.28)	33	(0.50)	16	(0.12)	9	(0.20)
Acute kidney injury	233	(0.46)	1536	(1.69)	1038	(2.38)	89	(1.34)	105	(0.81)	46	(1.02)
Post resuscitation state	24	(0.05)	40	(0.04)	440	(1.01)	37	(0.56)	38	(0.29)	800	(17.83)
Urological emergencies	28	(0.05)	27	(0.03)	2	(0.00)	2	(0.03)	8	(0.06)	0	(0.00)

ED, emergency department; ICU, intensive care unit; LTCH, long-term-care hospital; PTE/DVT, pulmonary thrombo-embolism/deep vein thrombosis.

**Table 6:** Procedures performed in the emergency departments of patients who were transferred from LTCHs.

	Preventable Visit		Non-Preventable Visit	
	*n*	%	*n*	%
	50,354		77,577	
Foley catheterization	20,311	(40.3)	1979	(2.6)
Placement of central venous catheter	6358	(12.6)	30,647	(39.5)
Nasogastric tube insertion	4597	(9.1)	873	(1.1)
Esophagogastroduodenoscopy	2979	(5.9)	343	(0.4)
Artificial ventilation	2349	(4.7)	1600	(2.1)
Invasive arterial blood pressure	2316	(4.6)	23	(0.0)
Transfusion	1626	(3.2)	3103	(4.0)
Tracheal intubation	1581	(3.1)	116	(0.1)
Hemodialysis	1392	(2.8)	244	(0.3)
Drug retention enema	1287	(2.6)	5449	(7.0)
Gastrostomy	937	(1.9)	57	(0.1)
Percutaneous transluminal angioplasty/others	607	(1.2)	2273	(2.9)
Thoracentesis	515	(1.0)	3	(0.0)
Pericardiocentesis	389	(0.8)	22	(0.0)
Upper gastrointestinal endoscopic bleeding control	352	(0.7)	6	(0.0)
Magnetic resonance imaging-brain	351	(0.7)	2389	(3.1)
Lumbar puncture	321	(0.6)	809	(1.0)
Percutaneous cholecystostomy	314	(0.6)	4618	(6.0)
Simple suture	299	(0.6)	1087	(1.4)
Cystostomy	296	(0.6)	1154	(1.5)
Closed thoracostomy	288	(0.6)	776	(1.0)
Percutaneous gastrostomy	285	(0.6)	296	(0.4)
Percutaneous transhepatic biliary drainage	169	(0.3)	124	(0.2)
Cardiopulmonary resuscitation	110	(0.2)	722	(0.9)
Invasive tracheostomy	55	(0.1)	9	(0.0)
Colonoscopic bleeding control	51	(0.1)	21	(0.0)
Decision to suspend life-sustaining treatment	45	(0.1)	0	(0.0)
Continuous hemodialysis	43	(0.1)	10,278	(13.2)
Endoscopic retrograde cholangiopancreatography	40	(0.1)	671	(0.9)
Endoscopic biliary or pancreatic drainage	40	(0.1)	104	(0.1)
Endoscopic treatment of esophageal or gastric variceal ligation	37	(0.1)	43	(0.1)
Percutaneous dilatational tracheostomy	6	(0.0)	2	(0.0)
Transtracheal catheter insertion and ventilation	4	(0.0)	7533	(9.7)
Endoscopic treatment of esophageal or gastric varices (Sclerotherapy)	2	(0.0)	196	(0.3)
Endoscopic treatment of upper gastrointestinal perforation	1	(0.0)	3	(0.0)
Dialysate exchange or catheter irrigation—conventional continuous ambulatory peritoneal dialysis	1	(0.0)	3	(0.0)
Break-in for chronic peritoneal dialysis	0	(0.0)	1	(0.0)

## Data Availability

The datasets used and/or analyzed during the current study are available from the corresponding author on reasonable request.

## Appendix A

**Table A1:** Comparison of common diagnosis by ED disposition results of LTCH group.

	Discharged			Admitted to GW			Admitted ICU			Transfer			Return to LTCH			Death		
	51,176	*n*	%	90,879	*n*	%	43,626	*n*	%	6642	*n*	%	12,952	*n*	%	4488	*n*	%
1	Pneumonia	2091	(4.1)	Pneumonia	13,763	(15.1)	Pneumonia	7590	(17.4)	Pneumonia	940	(14.2)	Pneumonia	1047	(8.1)	Cardiac arrest	744	(16.6)
2	Urinary tract infection	1883	(3.7)	Urinary tract infection	5097	(5.6)	Sepsis	2456	(5.6)	Urinary tract infection	259	(3.9)	Urinary tract infection	664	(5.1)	Pneumonia	607	(13.5)
3	End-stage kidney disease	1319	(2.6)	GI hemorrhage	3063	(3.4)	Urinary tract infection	1894	(4.3)	Aspiration pneumonia	212	(3.2)	Fever	353	(2.7)	Death	301	(6.7)
4	Anemia	1280	(2.5)	Aspiration pneumonia	2795	(3.1)	GI hemorrhage	1784	(4.1)	Fever	191	(2.9)	Dyspnea	340	(2.6)	Sepsis	222	(4.9)
5	Fever	1034	(2.0)	Fever	2729	(3.0)	Aspiration pneumonia	1761	(4.0)	Dyspnea	177	(2.7)	Anemia	316	(2.4)	Aspiration pneumonia	162	(3.6)
6	Gastroenteritis and colitis	952	(1.9)	Acute pyelonephritis	2185	(2.4)	Septic shock	1243	(2.8)	Sepsis	173	(2.6)	Aspiration pneumonia	283	(2.2)	Septic shock	157	(3.5)
7	Dyspnea	864	(1.7)	Cerebral infarction	1888	(2.1)	Cerebral infarction	1192	(2.7)	GI hemorrhage	145	(2.2)	End-stage kidney disease	246	(1.9)	Dyspnea	139	(3.1)
8	Gross hematuria	684	(1.3)	Melena	1789	(2.0)	Dyspnea	905	(2.1)	Septic shock	112	(1.7)	GI hemorrhage	225	(1.7)	GI hemorrhage	74	(1.6)
9	GI hemorrhage	680	(1.3)	Gastroenteritis and colitis	1483	(1.6)	Heart failure	898	(2.1)	Cerebral infarction	98	(1.5)	Melena	190	(1.5)	Unattended death	62	(1.4)
10	Abdominal pain, unspecific	672	(1.3)	Pleural effusion	1429	(1.6)	Acute renal failure	826	(1.9)	Heart failure	83	(1.2)	Malaise and fatigue	184	(1.4)	Urinary tract infection	53	(1.2)
